# Supplementary material for: Nanostructure Engineering of Metal–Organic Derived Frameworks: Cobalt Phosphide Embedded in Carbon Nanotubes as an Efficient ORR Catalyst
Source: Molecules. 2021 Nov 4;26(21):6672. doi: 10.3390/molecules26216672 (PMC8588548; doi:10.3390/molecules26216672)
Supplement: Supplementary file 1 [file molecules-26-06672-s001.zip › molecules-1444183-supplementary.pdf]

SUPPORTING INFORMATION

Nanostructure engineering of metal organic derived frameworks: Cobalt phosphide embedded carbon nanotubes as an efficient ORR catalyst

Syed Shoaib Ahmad Shah[1,2], Tayyaba Najam[3*], Costas Molochas[6], Muhammad Altaf Nazir[1], Angeliki Brouzgou[4], Muhammad Sufyan Javed[5], Aziz ur Rehman[1*], Panagiotis Tsiakaras [6,7.8*]

[1] Institute of Chemistry, the Islamia University of Bahawalpur 63100, Pakistan.

[2] Hefei National Laboratory for Physical Sciences at the Microscale, CAS Key Laboratory of Soft Matter Chemistry, Department of Chemistry, University of Science and Technology of China, Hefei, Anhui 230026, China.

[3] Institute for Advanced Study, Shenzhen University, Shenzhen 518060, China.

[4] Department of Energy Systems, Faculty of Technology, University of Thessaly, Geopolis, 41500, Larissa, Greece.

[5]School of Physical Science and Technology, Lanzhou University, Lanzhou 730000, P. R China.

[6] Laboratory of Alternative Energy Conversion Systems, Department of Mechanical Engineering, School of Engineering, University of Thessaly, Pedion Areos, 38834, Greece.

[7] Laboratory of Materials and Devices for Clean Energy, Department of Technology of Electrochemical Processes, Ural Federal University, 19 Mira Str., Yekaterinburg 620002, Russia.

[8] Laboratory of Electrochemical Devices Based on Solid Oxide Proton Electrolytes, Institute of High Temperature Electrochemistry, RAS, Yekaterinburg 620990, Russia.

*Corresponding authors: tayyabanajam@outlook.com, (T. Najam), azizypk@yahoo.com (A. Rehman), tsiak@uth.gr (P. Tsiakaras).

1. Figures and Tables

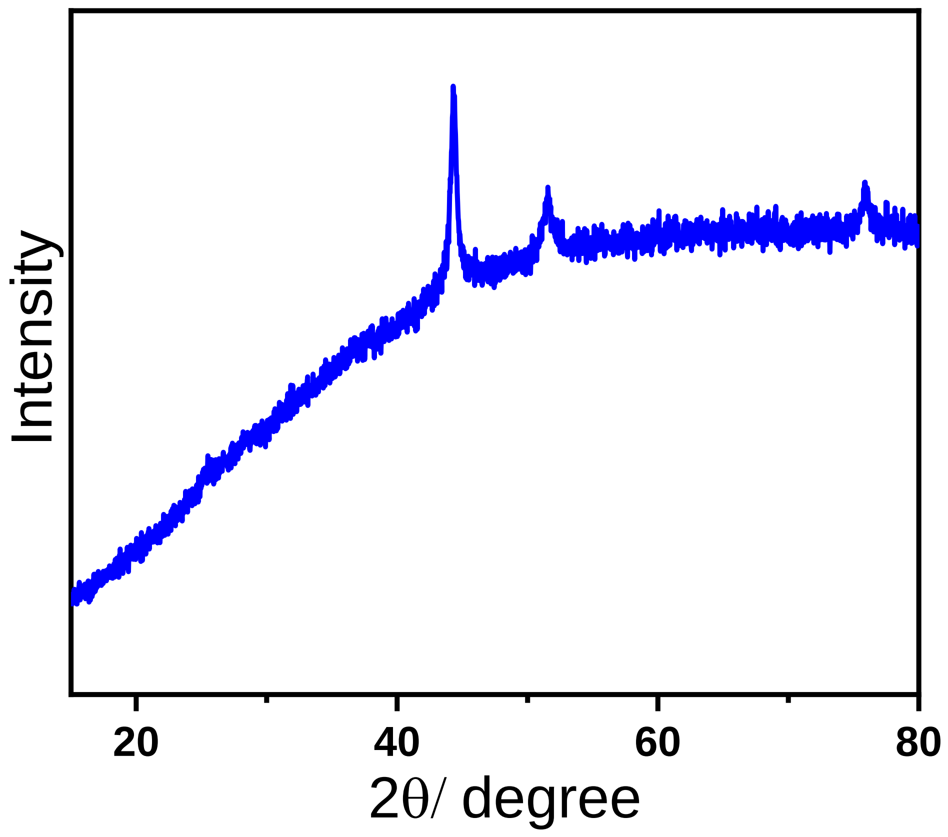

Figure S1: XRD pattern of Co-NC.

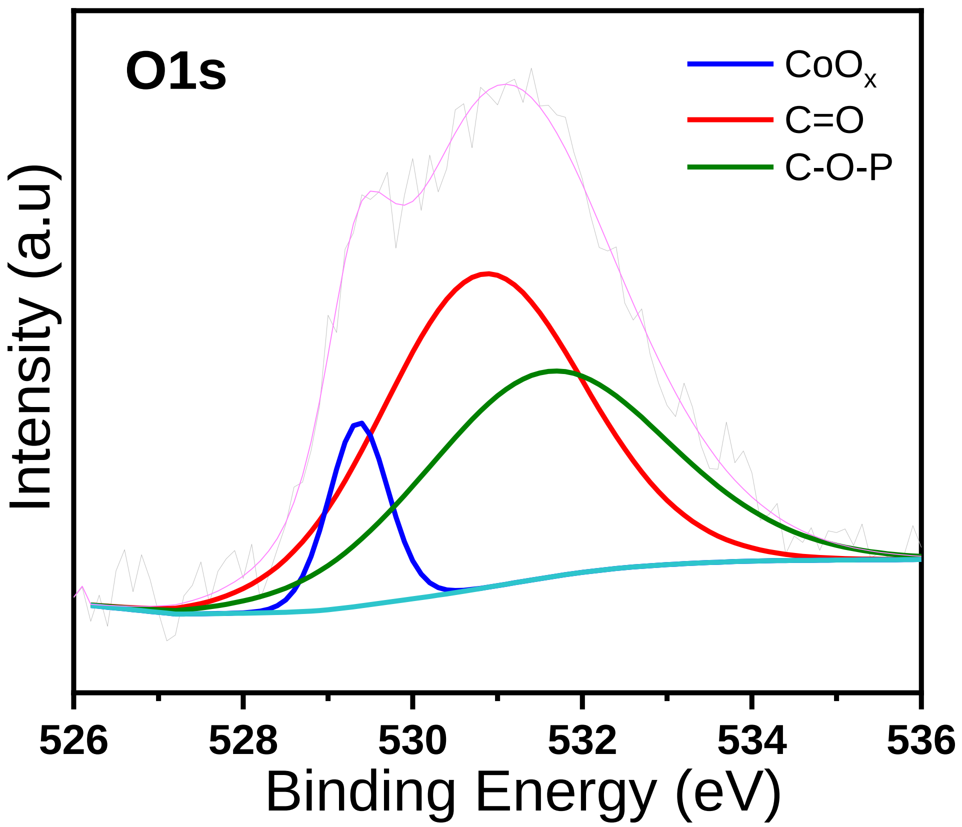

Figure S2: High resolution O1s XPS analysis of P-Co-CNTs.

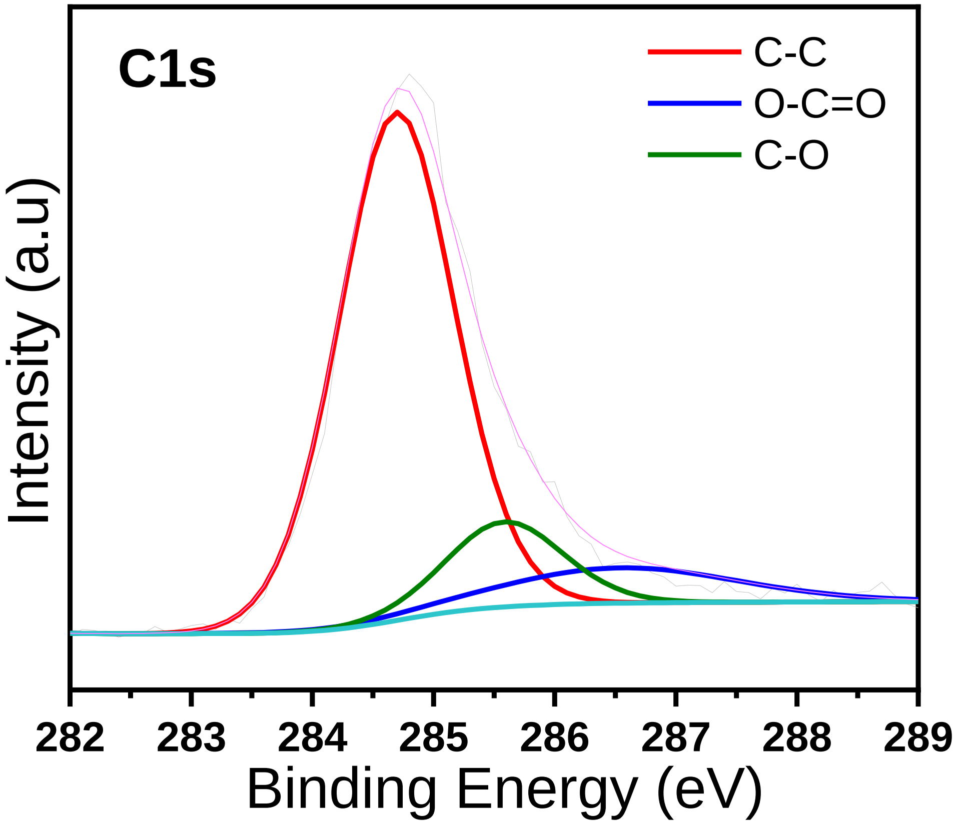

Figure S3: High resolution C1s XPS analysis of P-Co-CNTs.

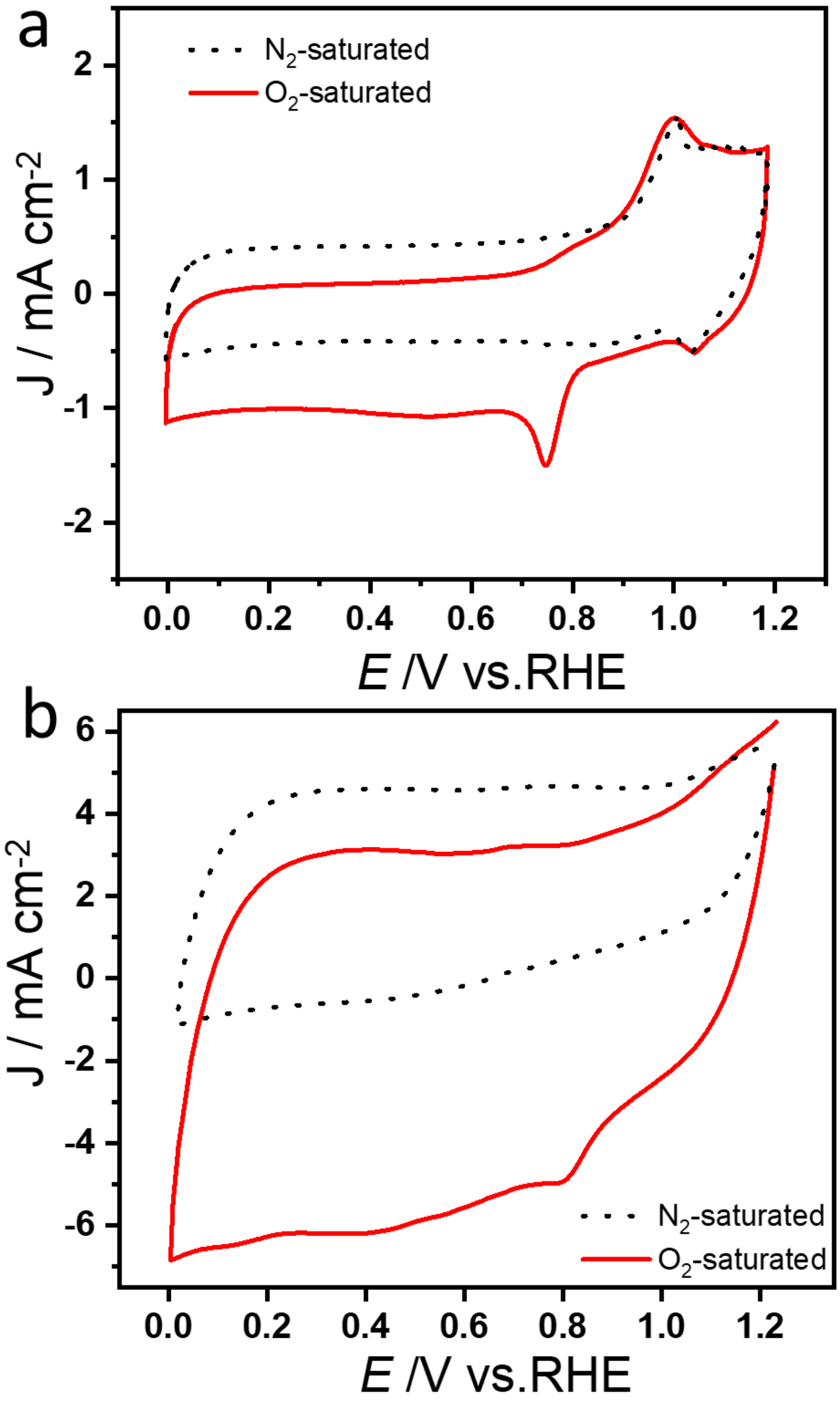

Figure S4: CV curves conducted under N_2 and O_2 saturated 0.1 M KOH solution; a) Co-CNTs, b) P-Co-CNTs.

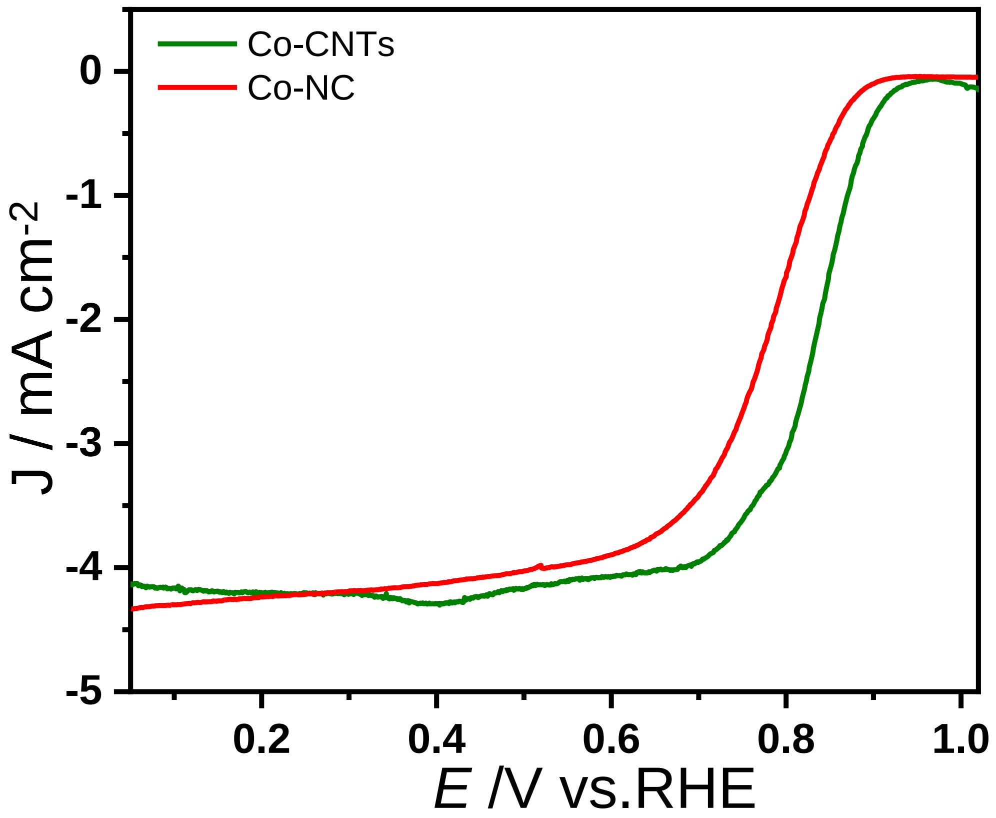

Figure S5: ORR-LSV curves conducted under O_2 saturated 0.1 M KOH solution.

Table S1. Elemental contents of P-Co-CNTs determined by XPS spectra.

**C** (*atm%*)	**N** (*atm%*)	**O** (*atm%*)	**P** (*atm%*)	**Co** (*atm%*)
91. 07	2. 16	3. 64	1. 61	1. 52

Table S2: Comparative study of ORR performance of the as-synthesized catalyst with reported catalysts under 0.1 M KOH solution vs. RHE.

**Catalyst**	**$E_{1/2}$ (V)**	**References**
P-Co-CNTs	*0.887*	*This work*
Co-N-C	0.87	[1]
Fe-N-C	0.88	[2]
$Co_{0.85}Se$@NC	0.817	[3]
Fe-N-C	0.86	[4]
NS/rGO-Co_4	0.84	[5]
Fe, Co–NC	0.85	[6]
Co_3O_4 /NCMTs	0.778	[7]
Co/N–PC	0.871	[8]
Co-N-C /PC	0.88	[9]
Co_9S_8/NC	0.82	[10]
CF-NG-Co	0.85	[11]
Co/N-PC	0.82	[12]
Co/N-C-800	0.74	[13]
CNT/N-PC	0.88	[14]
$CoS_{1.097}$–PC	0.81	[15]
CoS_2(400)/N,S-GO	0.79	[16]
Co/Co_3O_4-C	0.8	[17]

References:

[1] B.Y. Guan, L. Yu, X.W. Lou, Formation of Single-Holed Cobalt/N-Doped Carbon Hollow Particles with Enhanced Electrocatalytic Activity toward Oxygen Reduction Reaction in Alkaline Media, Advanced Science, 4 (2017) 1700247.

[2] T. Liu, P. Zhao, X. Hua, W. Luo, S. Chen, G. Cheng, An Fe-N-C hybrid electrocatalyst derived from a bimetal-organic framework for efficient oxygen reduction, Journal of Materials Chemistry A, 4 (2016) 11357-11364.

[3] T. Meng, J. Qin, S. Wang, D. Zhao, B. Mao, M. Cao, In situ coupling of Co0.85Se and N-doped carbon via one-step selenization of metal–organic frameworks as a trifunctional catalyst for overall water splitting and Zn–air batteries, J. Mater. Chem. A, 5 (2017) 7001-7014.

[4] S.H. Ahn, X. Yu, A. Manthiram, “Wiring” Fe-Nx-Embedded Porous Carbon Framework onto 1D Nanotubes for Efficient Oxygen Reduction Reaction in Alkaline and Acidic Media, Advanced Materials, 29 (2017) 1606534.

[5] N. Wang, L. Li, D. Zhao, X. Kang, Z. Tang, S. Chen, Graphene Composites with Cobalt Sulfide: Efficient Trifunctional Electrocatalysts for Oxygen Reversible Catalysis and Hydrogen Production in the Same Electrolyte, Small, 13 (2017) 1701025.

[6] J. Xi, Y. Xia, Y. Xu, J. Xiao, S. Wang, (Fe,Co)@nitrogen-doped graphitic carbon nanocubes derived from polydopamine-encapsulated metal-organic frameworks as a highly stable and selective non-precious oxygen reduction electrocatalyst, Chem. Commun., 51 (2015) 10479-10482.

[7] B. Wang, L. Xu, G. Liu, P. Zhang, W. Zhu, J. Xia, H. Li, Biomass willow catkin-derived Co3O4/N-doped hollow hierarchical porous carbon microtubes as an effective tri-functional electrocatalyst, J. Mater. Chem. A, 5 (2017) 20170-20179.

[8] B. You, N. Jiang, M. Sheng, W.S. Drisdell, J. Yano, Y. Sun, Bimetal–Organic Framework Self-Adjusted Synthesis of Support-Free Nonprecious Electrocatalysts for Efficient Oxygen Reduction, ACS Catalysis, 5 (2015) 7068-7076.

[9] P. Yin, T. Yao, Y. Wu, L. Zheng, Y. Lin, W. Liu, H. Ju, J. Zhu, X. Hong, Z. Deng, G. Zhou, S. Wei, Y. Li, Single Cobalt Atoms with Precise N-Coordination as Superior Oxygen Reduction Reaction Catalysts, Angewandte Chemie International Edition, 55 (2016) 10800-10805.

[10] H. Hu, L. Han, M. Yu, Z. Wang, X.W. Lou, Metal-organic-framework-engaged formation of Co nanoparticle-embedded carbon@Co9S8 double-shelled nanocages for efficient oxygen reduction, Energy & Environmental Science, 9 (2016) 107-111.

[11] Z. Pei, Z. Tang, Z. Liu, Y. Huang, Y. Wang, H. Li, Q. Xue, M. Zhu, D. Tang, C. Zhi, Construction of a hierarchical 3D Co/N-carbon electrocatalyst for efficient oxygen reduction and overall water splitting, J. Mater. Chem. A, 6 (2018) 489-497.

[12] J.-N. Zheng, S.-S. Li, X. Ma, F.-Y. Chen, A.-J. Wang, J.-R. Chen, J.-J. Feng, Green synthesis of core-shell gold-palladium@palladium nanocrystals dispersed on graphene with enhanced catalytic activity toward oxygen reduction and methanol oxidation in alkaline media, Journal of Power Sources, 262 (2014) 270-278.

[13] Y. Su, Y. Zhu, H. Jiang, J. Shen, X. Yang, W. Zou, J. Chen, C. Li, Cobalt nanoparticles embedded in N-doped carbon as an efficient bifunctional electrocatalyst for oxygen reduction and evolution reactions, Nanoscale, 6 (2014) 15080-15089.

[14] D. Geng, N. Ding, T.S.A. Hor, Z. Liu, X. Sun, Y. Zong, Potential of metal-free "graphene alloy" as electrocatalysts for oxygen reduction reaction, Journal of Materials Chemistry A, 3 (2015) 1795-1810.

[15] F. Bai, H. Huang, C. Hou, P. Zhang, Porous carbon-coated cobalt sulfide nanocomposites derived from metal organic frameworks (MOFs) as an advanced oxygen reduction electrocatalyst, New Journal of Chemistry, 40 (2016) 1679-1684.

[16] P. Ganesan, M. Prabu, J. Sanetuntikul, S. Shanmugam, Cobalt Sulfide Nanoparticles Grown on Nitrogen and Sulfur Codoped Graphene Oxide: An Efficient Electrocatalyst for Oxygen Reduction and Evolution Reactions, ACS Catal., 5 (2015) 3625-3637.

[17] A. Aijaz, J. Masa, C. Rösler, W. Xia, P. Weide, A.J.R. Botz, R.A. Fischer, W. Schuhmann, M. Muhler, Co@Co3O4 Encapsulated in Carbon Nanotube-Grafted Nitrogen-Doped Carbon Polyhedra as an Advanced Bifunctional Oxygen Electrode, Angewandte Chemie International Edition, 55 (2016) 4087-4091.
